# Supplementary material for: MetaRibo-Seq measures translation in microbiomes
Source: Nat Commun. 2020 Jun 29;11:3268. doi: 10.1038/s41467-020-17081-z (PMC7324362; doi:10.1038/s41467-020-17081-z)
Supplement: Supplementary file 10 — Supplementary Data 7 [file 41467_2020_17081_MOESM10_ESM.zip › File2/Confidence_VeryHigh_Taxonomy/128136_out.krona.html]

Javascript must be enabled to view this page.

members
magnitude
magnitudeUnassigned
count
unassigned
taxon
rank

128136\_out


SRS146812\_contig\_number\_contig-100\_2279.312620
1
4

1

SRS014855\_contig\_number\_contig-100\_41473.41474

2
2
superkingdom

1239
phylum
2

class
186801
1

order
186802
1

1
541000
family


SRS104400\_contig\_number\_8975
species
39492
1

1
1263003
species

SRS143466\_contig\_number\_29113
